# The Impact of Coronavirus Infection on Health-Related Quality of Life in Amateur CrossFit Athletes

**DOI:** 10.3390/ijerph192416409

**Published:** 2022-12-07

**Authors:** Sebastian Szajkowski, Jarosław Pasek, Michał Dwornik, Grzegorz Cieślar

**Affiliations:** 1 Faculty of Medical Sciences, Medical University of Mazovia in Warsaw, 8 Rydygiera St., 01-793 Warsaw, Poland; 2 Faculty of Health Sciences, Jan Długosz University in Częstochowa, 13/15 ArmiiKrajowej St., 42-200 Czestochowa, Poland; 3 Department of Internal Medicine, Angiology and Physical Medicine, Faculty of Medical Sciences in Zabrze, Medical University of Silesia in Katowice, 15 Stefana Batorego St., 41-902 Bytom, Poland

**Keywords:** quality of life, COVID-19, CrossFit, sports activity, amateur athletes, coronavirus infection

## Abstract

Background: The aim of this study was to assess how the disease, developing over the course of coronavirus infection, affects the quality of life of athletes practicing amateur sports who are not burdened with comorbidities. Methods: The study included 102 amateur CrossFit athletes (54 (53%) women and 48 (47%) men) who had been infected with SARS-CoV-2, but were not hospitalized. The training experience of the respondents ranged from 1.5 to 15 years (average: 6.7 ± 3.8 years). The quality of life was assessed with EQ-5D-5L and Clinical COPD Questionnaire (CCQ), which was used to assess the quality of life specific to the respiratory system, and the severity of dyspnea was assessed using the MRC questionnaire. Results: The training experience did not differ statistically significantly between men and women (*p* = 0.595). The quality of life in men according EQ-5D-5L was statistically significantly higher than in the case of women (0.979 ± 0.028 vs. 0.942 ± 0.073 (*p* < 0.001), respectively), and in EQ-VAS it was significantly higher in men than in women (85.64 ± 10.4 vs. 72.5 ± 19.36 points (*p* < 0.001)). The assessment of dyspnea by means of mMRC showed its higher intensity in women than in men. The differences were not statistically significant (*p* = 0.195). In men, a significantly lower result of the quality of life was noted in the CCQ questionnaire: 0.71 ± 0.57 vs. 1.14 ± 0.84 points (*p* = 0.009). Conclusions: The HRQOL, which surveyed amateur CrossFit after COVID-19, was higher in men than in women. People practicing strength- and strength-endurance-based sports rated their quality of life as the highest. Most of the subjects observed a slight intensification of dyspnea. The findings can be used for future healthcare measures to be applied in the population of CrossFit athletes.

## 1. Introduction

The COVID-19 pandemic may have a potentially serious impact on mental health and can increase the risk of anxiety, depression, and post-traumatic disorders, especially in patients with COVID-19 or individuals who have had contact with COVID-19 patients. The outbreak of the COVID-19 pandemic has caused significant disruptions to people’s lives. To slow the spread of the disease, lockdown measures have been put in place that limit people’s ability to leave their homes and interact with others. How these measures impact people’s mental health is a major public health concern [1,2].

The COVID-19 pandemic has necessitated the closure of sports centers and public spaces that allow sports to be practiced, as part of social distancing measures adopted around the world. This has limited the ability of many people to conduct and maintain physical activity at the previous level. Studies have shown that quarantine can cause a significant decrease in physical activity levels, despite the use of individual training processes [3,4]. Facer-Childs E.R. et al. surveyed elite and sub-elite athletes (n = 565) across multiple sports. Significant disruptions were reported for all lifestyle factors, including social interactions, physical activity, sleep patterns, and mental health. Training frequency and duration decreased significantly. Importantly, the changes in training and sleep-related factors were associated with mental health outcomes [5]. Similar results were obtained by Italian researchers Casali N. et al., who showed statistically significant differences in the level in Physical Activity (PA) changes from pre-lockdown to lockdown time and who examined the relationships concerning lockdown PA, quality of life (mental and physical health), motivation to exercise, psychological distress, intolerance of uncertainty, and body dissatisfaction [6]. Of course, all the above factors have had a negative effect on both the physical and mental condition of the subjects. Recent studies pointed out the protective role of physical activity and competition during lockdown [3,4]. Lockdown measures included: travel restrictions, the mandatory closure of schools, as well as non-essential commercial activities and industries. People were required or asked to stay at home and socially isolate themselves to prevent being infected [7]. At the individual level, epidemics are associated with a wide range of psychiatric comorbidities, including anxiety, panic, depression, and trauma-related disorders [8]. Grubic N. et al. also emphasize an imminent need to understand how academic institutions, sports organizations and healthcare systems can collaborate in their response to the COVID-19 crisis, so that negative psychological outcomes can be mitigated and athletes can return to competing [9].

Among patients with active lifestyles, not burdened with comorbidities, a milder course of the disease is observed as a consequence SARS-CoV-2 infection. Most of such patients do not require hospitalization and have a relatively mild course of the disease [10]. At the same time, there are concerns about the potential, long-term consequences of the disease, which determine the worsened mental state of the sick and convalescents [11].

The impact of COVID-19 infection and post-COVID syndrome on the quality of life of ambulatory patients may be significantly underestimated, as most studies assessing the quality of life over the course of COVID-19 infection focus on hospitalized patients [12,13]. A decidedly smaller number of studies concern cases that do not require hospitalization, and their results indicate the need for further research in that area [14].

Recently, Quality of Life (QoL) assessment has been of interest to many medical disciplines. Some studies emphasize the present overall health condition; alternatively, the assessment concerns the period following the disease the person suffered from. For a human being, the quality of life is related to a successful family life, work, health, the ability to deal with difficult situations, if any, the motivation to act and the sense of fulfillment. The quality of life depends on the health condition and includes ingredients such as psychological and somatic symptoms, physical activity, the sense of satisfaction and sexual functions. The quality of life in psychology is recognized as subjective experiences of an individual that result from everyday life (satisfaction with life, mental well-being) [15,16].

The World Health Organization (WHO) defines QoL as “an individual’s perception of their position in life in the context of the culture and value systems in which they live and in relation to their goals, expectations, standards and concerns”. In particular, health-related quality of life (HRQOL) is an evaluation of QoL and its relationship to health. Different tools are available to measure HRQOL, as it has multidimensional components. It includes physical, psychological, functional, and social domains related to a person’s perception of QoL affected by that person’s health status [15].

The most common questionnaires to assess HRQOL are the 36-item short-form (SF-36) survey or the EuroQoL 5-domain 5-level (EQ-5D-5L) tool [15,17].

## 2. Aim of the Study

The aim of the study was to assess how the developing of disease over the course of coronavirus infection affects the quality of life of people practicing amateur sports, who are not burdened with comorbidities. It has been hypothesized that the widely understood Health-Related Quality of Life (HRQOL) in the case of COVID-19 is influenced not only by the condition of the respiratory tract, but also by limiting the fulfillment of functional needs and the person’s mental state. To achieve this goal, a study was carried out in which several different questionnaires assessing the quality of life were used.

## 3. Material and Methods

All participants gave written informed consent to participate in the study. The Medical University of Mazovia was approved by the local ethics committee and the guidelines outlined in the Declaration of Helsinki were followed (MUM/2021/39).

The participants were recruited using an online survey platform (Google). A total of 102 people training amateur CrossFit (54 (53%) women and 48 (47%) men) took part in the study. The training period of people qualified for the study was in the range of: 1.5–15 years, with an average training period of 6.7 ± 3.8 years. The mean age of participants was 34.6 ± 10.5 years. The average BMI was 22.3 ± 2.5 (kg/m^2^) for women and 26.2 ± 2.5 (kg/m^2^) for men. The BMI index in 57 (55.8%) of the respondents indicated normal body weight and was in the range of 18.5–24.9 kg/m^2^. In 45 (44.1%) subjects, the values indicated that they were overweight: 25.0–29.9 kg/m^2^.

The inclusion criteria for the study were as follows: infection with the SARS-CoV-2 virus confirmed with a positive test result, age in the range of 20–60 years, active amateur sports (CrossFit and CrossFit with additional sports activity), no chronic diseases, no smoking, no hospitalization over the course of infection and illness, informed and voluntary consent to participate in the study.

In order to analyze the collected data, the following age ranges of the participants of the study were distinguished: 20–29 years, 30–39 years, 40–49 years, and 50–60 years. Similarly, the participants were divided into groups according to the number of years relating to training experience: 1–5 years, 6–10 years and 11–15 years.

The research tool was a questionnaire that was sent to 12 affiliated CrossFit clubs in Poland. The study was attended by amateurs training CrossFit and CrossFit with other sports. The sports additionally practiced by the respondents included: cycling, gym exercises, running, and Nordic Walking. The study was conducted from October 2021 to January 2022. A total of 131 questionnaires completed by respondents were received, and 102 of them complied with the inclusion criteria.

The first part contains questions about the basic demographic data of the respondents. In the second part, the quality of life was assessed using the EQ-5D-5L questionnaire and the Clinical COPD Questionnaire (CCQ), the latter being used to assess the quality of life specific to the respiratory system, and with the use of modified mMRC questionnaire to assess the severity of dyspnea [18,19].

## 4. Generic Quality of Life

The EQ-5D-5L questionnaire was used to assess the quality of life (EuroQol Research Foundation). The questionnaire assesses five dimensions (dimensions of everyday life): Mobility (MO), Self-care (SC), Usual activities (UA), Pain/Discomfort (PD) and Anxiety/Depression (AD). The answers are categorized in five levels, ranking as follows: grade 1: no problems, grade 2: slight problems, grade 3: moderate problems, grade 4: severe problems, grade 5: extreme problems/inability to cope. Combining the dimension-specific levels across the five dimensions provides distinct health states (e.g., 21,111, meaning slight problems in the mobility dimension and no problems in any of the other dimensions). The questionnaire defines 3125 health states. Based on these five-digit codes, an index score (EQ-Index) is provided (according to the preferences of the general population of a country/region), which ranges from −0.590 (worst quality of life) to 1 (best quality of life), with a score <0 representing a health state worse than death. The second part of the assessment comprised the visual analogue EQ-VAS scale (EuroQol Visual analogue scale). EQ-VAS contains a scale from 0 to 100, where 0 is the worst imaginable health condition and 100 is the best imaginable health condition [20,21].

## 5. The Clinical COPD Questionnaire (CCQ)

The validated Dutch version of the CCQ (Clinical COPD Questionnaire) was used to assess respiratory-specific quality of life. The CCQ is a 10-item assessment tool divided into three domains: symptoms (dyspnea, cough, and phlegm), functional state (limitations in different activities of daily life due to respiratory symptoms) and mental state (feeling depressed and having concerns about breathing). The questions were rated on a seven-point scale, ranging from 0: never/not limited at all, to 6: all the time/totally limited or unable to. The main outcomes are the CCQ total scores (sum of all items divided by 10) and the mean scores of the three separate dimensions. The higher the obtained result, the lower the quality of life [22,23].

## 6. Respiratory-Specific Quality of Life

The study used a modified mMRC questionnaire (modified British Medical Research Council questionnaire). It includes a five-point (1 to 5) scale for assessing the severity of dyspnea, in which grade 1 stands for dyspnea occurring only with strenuous exercise, grade 2: dyspnea when hurrying or walking up slightly uphill a slight hill, grade 3: the person walks slower than people of the same age because of dyspnea or has to stop to catch breath when walking at her own pace, grade 4: the person stops for breath after walking 100 yards or after a few minutes, step 5: too dyspneic to leave home or breathless when dressing [22,23].

## 7. Statistical Analysis

The statistical analysis of the data collected was performed using the Statistica 13 (StatSoft, Kraków, Poland) software package. The results of the study are presented using mean values and standard deviation. The nature of the distribution of the investigated quantitative variables was assessed using the Shapiro–Wilk test. Non-normal distributions of data were found. The Mann–Whitney and Kruskal–Wallis U tests were used to test the statistical significance of the differences in the studied parameters between individual subgroups. Qualitative variables were assessed using the chi-square test. The *p* value of <0.05 was adopted as the level of statistical significance.

## 8. Results

The mean EQ-5D-5L index-score was 0.959 ± 0.059 and ranged from 0.509 to 1.000. In the group of men, the mean EQ-5D-5L index-score was 0.979 ± 0.028 and was higher than in the group of women, at 0.942 ± 0.073 in the latter. This difference was statistically significant (*p* < 0.001), but it was not clinically meaningful. The highest values in the EQ-5D-5L index-score were obtained in people training CrossFit only and CrossFit with a gym (0.986 ± 0.024 and 0.961 ± 0.087, respectively), and the lowest values were noted for CrossFit with Nordic Walking (0.932 ± 0.047). The differences observed in the EQ-5D-5L index-score values, between the groups of people practicing the sport disciplines included in the study, were statistically significant (*p* = 0.0016). There were no statistically significant differences between the mean values of the EQ-5D-5L index-score in separate groups, which would occur due to the age range, BMI index or training experience (Table 1).

The mean EQ-VAS score was 78.68 ± 17.05 and its values ranged from 20 to 100. There were statistically significant differences (*p* = 0.000155) in mean EQ-VAS score values between the group of women and that of men. Men obtained higher results: 85.64 ± 10.4 and 72.5 ± 19.36, respectively. Among people training additionally in the gym and cycling, the highest values of EQ-VAS score were recorded: 86.52 ± 17.44 and 84.92 ± 8.78, respectively. The lowest values (68.18 ± 17.26) were observed in people practicing Nordic Walking. The differences in the mean EQ-VAS score values between the respondents practicing sports included in the study reached the level of statistical significance (*p* = 0.0013). There were no statistically significant differences between the mean EQ-VAS score values in the groups distinguished by age range, BMI index, or training experience (Table 1).

Table 2 presents the results of the assessment of quality of life specific for the respiratory system, according to the CCQ questionnaire. The mean CCQ Total score was 0.94 ± 0.75 and ranged from 0 to 3.8. Lower mean values of the CCQ Total score, indicating a higher quality of life, were observed among men, and amounted to 0.71 ± 0.57. In women, this mean was 1.14 ± 0.84. These differences were statistically significant (*p* = 0.009). Similar observations were made as concerns the mean CCQ Functional state results for the group of women and that of men, amounting to 1.03 ± 0.9 and 0.42 ± 0.5, respectively. These differences also turned out to be statistically significant (*p* < 0.001). There were no differences in the level of statistical significance between men and women in the obtained mean values of the following parameters: CCQ Symptoms and CCQ Mental state. In terms of CCQ Total score, CCQ Symptoms and CCQ Functional state, statistically significant differences in the quality of life were noted in relation to the sports practiced by the respondents. The levels of statistical significance were as follows: for the CCQ Total score—(*p* < 0.001), for CCQ Symptoms—(*p* = 0.007), and for CCQ Functional state–(*p* < 0.001), respectively. The lowest values denoting the best quality of life concerned people training: CrossFit and at the gym, CrossFit and cycling, CrossFit only. The sense/feeling of a lower quality of life was demonstrated by the respondents practicing CrossFit and Nordic Walking, as well as CrossFit and running. The differences between sports in the CCQ Mental state parameter did not reach the level of statistical significance. Only with regard to this parameter, statistically significant (*p* = 0.0381) differences were noted between the groups distinguished in terms of the length of training history. The highest quality of life was recorded in people training for 1–5 years (0.71 ± 1.02); it was rated lower by people training longer. There were no statistically significant differences between the groups in terms of age and BMI in any of the tested CCQ parameters (Table 2).

Table 3 shows the distribution of study participants by subgroups, according to the severity of dyspnea. The degree of severity of dyspnea in all respondents ranged between 1 and 3 according to of the mMRC questionnaire, and none of the participants assessed the severity of dyspnea at the level corresponding to grade 4 or 5. Most subjects, as many as 65 people (63.72%), rated the severity of dyspnea as grade 1. Men more often indicated severity of dyspnea as grade1 (70.83%), compared to women (57.41%). Only one man assessed his dyspnea as grade 3. The differences between the genders were not statistically significant (*p* = 0.195). The younger the respondent’s age, the more often s/he indicated lower severity of dyspnea, with participants aged 40–49 years constituting the highest percentage of the study participants (76%). None of the members of this group rated the intensity of their dyspnea as grade 3 or higher. The differences in the severity of dyspnea between the age groups of participants were not statistically significant (*p* = 0.307). Similarly, no statistically significant differences were found between the ranges related to the BMI value (*p* = 0.763).

Only two people from each training period assessed the severity of dyspnea as grade 3. Most (68.63%) respondents in the 1–5 years training period indicated dyspnea at the lowest grade of 1. The differences observed did not reach the level of statistical significance (*p* = 0.595). Similarly, statistically insignificant differences were noted in the severity of dyspnea between persons practicing sports included in the study (*p* = 0.198). However, in the vast majority of cases, the severity of dyspnea according to the mMRC questionnaire was indicated by people training CrossFit and additionally in the gym (17 cases (89.47%)), and those training only CrossFit (22 cases (68.75%)).

## 9. Discussion

The presented results of research concerning the assessment of the quality of life of amateurs training CrossFit prove that athletes practicing CrossFit feel the consequences of a history of COVID-19 disease, just like athletes practicing other sports. This is confirmed by the results of studies by other authors [24,25].

The quality of life of athletes during the COVID-19 pandemic was also assessed by Jia L et al., who reviewed the databases (PubMed, Embase, and Cochrane Library), taking into account the available articles (level of evidence: 4) aimed at assessing mental health and emotional well-being of athletes during the COVID-19 pandemic. A total of 35 studies of athletes from around the world were included in the final analysis. The results of the analysis showed that athletes reported poorer mental and emotional health during the COVID-19 pandemic, although these effects were mitigated by home-based training programs and training camps organized during the quarantine. In this case, the female gender was associated with an increased risk of poorer mental health outcomes [26].

On the other hand, Cosma GA et al. assessed the quality of life of 249 athletes during the COVID-19 pandemic. The results of their study showed significant differences in the severity of anxiety related to COVID-19, depending on the type of sports practiced. The authors, as in the case of our study, did not note statistically significant differences in terms of sex or age, and the quality of life of the assessed athletes. However, they showed a significant influence of social isolation and quarantine on the emotional well-being of athletes [27].

In another analysis of databases (PubMed, Scopus, and Embase) carried out by Jurecka A. et al., assessing the impact of the pandemic on physical activity, mental state and quality of life of professional athletes, the authors also showed the negative effects of the pandemic manifested, among others, by a decrease in general fitness and the number of days and hours of training, as well as an increase in the incidence of negative emotions (stress, fatigue, depression) and deterioration in the quality of sleep. In this case, the subjects of female gender experienced negative emotions more often than men [28].

Condel E et al. assessed the impact of the health crisis related to the COVID-19 pandemic in 130 Spanish competitive athletes in terms of the intensity of practicing sports, quality of life and emotional state, and attempted to identify the profile of athletes with the greatest difficulties during COVID-19 and after quarantine. The study used a questionnaire containing the International Physical Activity Questionnaire (IPAQ), the SF-12 Health Questionnaire, perceived stress (Short-PSS), and mood states (POMS 29 points). All participants showed a significantly reduced perception of their health status and physical performance. This was especially true of women with severe limitations in training intensity, attention, and motivation, as well as a moderately negative emotional state and the elevated level of stress during quarantine [29].

In our study, we noticed differences in the assessment of the quality of life among people training amateur CrossFit alone and groups of people declaring simultaneous practice of an additional sport: cycling, gym exercises, running, or Nordic Walking. These differences may result, inter alia, from the specificity of the place where sports are practiced, which should be an important factor in diversifying training in the post-pandemic period. The diversified impact of environmental changes on the trainings carried out during the pandemic is also confirmed in other scientific studies [30,31]. It is difficult to indicate which athletes are more likely to develop complications due to COVID-19 [32]. It is known, however, that the absence of physical activity, also in people who previously practiced sports regularly, is the main risk factor for a more severe course of infectious diseases. In the case of COVID-19, it may contribute to the development of the post-COVID syndrome [33]. The subjective feeling of deteriorating quality of life after suffering from COVID-19 disease, as shown by many studies, depends on environmental and social factors, and the assessment of the quality of life is influenced not only by the lack of regular training, but also by the general public mood [34].

Literature data show that the possibility of training outdoors has a significant impact on the quality of life [35]. This is confirmed by the results of our research. People who, while practicing physical activity, additionally stayed in the open space rated their quality of life higher. It concerned an amateur group training CrossFit together with Nordic Walking. Outdoor training can therefore be an important part of CrossFit training in the lockdown and post-lockdown period.

The results of our survey show that the assessment of the quality of life was not statistically influenced by age, BMI, or training experience. According to Kelly AL. et al., the subjective assessment of the quality of life may significantly depend on the possibility of exercising at home during on-line training [36].

The presented study also shows that the groups of people training CrossFit with Nordic Walking and CrossFit with running rated the level of their respiratory efficiency the lowest. This may be related to the type of additional aerobic activity that requires the proper functioning of the respiratory tract. Infection with COVID-19 reduces the ability to absorb oxygen [37]. The sense of quality of life related to breathing in people training longer was statistically significantly deteriorated/worse, as compared to people with a shorter training experience. According to Dors H. et al., training history is associated with the ability to adapt to a greater oxygen debt and better absorption of oxygen [38].

As a result of COVID-19 disease, respiratory parameters deteriorate. There are reports in the literature on the subject which indicate that people with better physical performance before the disease suffer less profoundly from the disease, and their respiratory efficiency after the disease should be relatively higher [29]. Therefore, it seems that people with longer training experience, with better training efficiency, should rate their respiratory efficiency after COVID-19 more highly. It turns out, however, that despite the generally better respiratory efficiency, the group of people with longer training experience and better endurance level perceived the deterioration of their aerobic fitness more clearly, which had a direct impact on a lower declared quality of life. Hence, in athletes with longer training experience and a higher degree of training, it is necessary to pay particular attention to their emotional state when returning to full-scale training.

In people systematically practicing sports, dyspnea after COVID-19 infection is usually more or less severe [39]. Our study participants did not report any significant increase in dyspnea level. There were no statistically significant differences in the assessment of the severity of dyspnea between the groups, in terms of: sports practiced, training experience, BMI and gender. Respiratory capacity, which is at such a relatively high level after being infected with COVID-19 in the present study, can be explained by the regular physical effort of all its participants, regardless of the sports practiced. Another reason for such results may be the nature of the sports practiced by the sportsmen studied and their non-competitive level of advancement. People training CrossFit as amateurs potentially have lower exercise load than those training typical endurance sports at the competitive level, in which one quarter of them experience increased symptoms of exercise dyspnea [40].

Deterioration of the quality of life has been observed in all people after contracting COVID-19 [33]. This fact should be taken into account when people of all ages return to training, also in those athletes whose course of disease was mild and no complications were found in the long-term follow-up [41,42].

While isolation is an essential public health measure, the results show that it has negative effects on physical activity in a way that endangers health. More detailed data analyses can help design interventions to alleviate negative lifestyle behaviors that emerged during the COVID-19 pandemic. This also applies to the recommendations regarding physical activity in athletes (including amateur athletes), which should be in line with the current guidelines and standards for pre-exercise testing. More specific advice will also be required, including an assessment of sports qualifications and specific exercise programs to get one back into sport. Due to the current uncertainty about the long-term course of SARS-CoV-2 infection or COVID-19 disease, long-term observation seems to be necessary. Athletes should also continue to monitor and record their own physical and psychological indicators of health.

## 10. Limitations of the Study

The authors did not collect information about the time that passed between questionnaire completion to COVID-19 infection. The period when the participants were infected with SARS-CoV-2 was unknown. The data concerning the body composition of CrossFit athletes is also absent. Those who were diagnosed earlier on may have recovered fully and thus not reported a reduced HRQOL, compared to those who were recently infected (and thus more recently faced the physical/mental consequences, such as time missed in training). There are two prominent biases in this study: the selection bias (volunteer bias) and that of misclassification (from self-reported measures). In addition, questionnaires are characterized by a lack of control over external factors that accompany the research, no possibility to verify the identity of the respondents, superficiality, inability to deepen the analysis of the problem, and absence of the possibility of direct contact with the respondent. They may have impacted the study findings. The authors did not include a control group to compare with (athletes without a prior COVID-19 infection). This would further help us to understand whether COVID-19 infection is a significant contributor to HRQOL.

## 11. Conclusions

In conclusion, presented above is the assessment of respiratory-specific and generic quality of life in non-hospitalized amateur CrossFit athletes with confirmed SARS-CoV-2 infection using validated measures. Hence, we can derive important insights into the evaluation of quality of life in this population. The quality of life of amateur CrossFit athletes depends on the type of complementary training, and strength and endurance athletes rated their quality of life the highest. Most of the subjects observed a slight intensification of dyspnea. In the mental sphere, people with longer training experience coped worst with the effects of the disease. The assessed quality of life of the surveyed amateur CrossFit practitioners after being infected with COVID-19 was higher in men than in women. The article provides important evidence concerning HRQOL during the COVID-19 pandemic. Our findings can be used for future healthcare measures among the population of CrossFit athletes.

## Figures and Tables

**Table 1:** The results of the assessment of the quality of life according to the EQ-5D-5L index and EQ-VAS questionnaire with the statistical evaluation.

	General Characteristics	EQ-5D-5L Index-Score		EQ-VAS Score	
	n (%)	Mean ± SD	*p*-Value	Mean ± SD	*p*-Value
**Total**	102 (100)	0.959 ± 0.059		78.68 ± 17.05	
**Gender**					
**Female**	54 (52.9)	0.942 ± 0.073	**<0.001**	72.5 ± 19.36	**<0.001**
**Male**	48 (47)	0.979 ± 0.028		85.64 ± 10.4	
**Age (years)**					
**20–29**	39 (38.2)	0.964 ± 0.042	0.315	80.15 ± 16.89	0.717
**30–39**	28 (27.4)	0.951 ± 0.092		77.75 ± 18.75	
**40–49**	25 (24.5)	0.969 ± 0.035		77.32 ± 18.66	
**50–60**	10 (9.7)	0.940 ± 0.043		79 ± 7.4	
**BMI (kg/m^2^)**					
**18.5–24.9**	57 (55.8)	0.957 ± 0.039	0.105	75.68 ± 19.70	0.156
**25.0–29.9**	45 (44.1)	0.961 ± 0.077		82.48 ± 12.11	
**Training history (years)**					
**1–5**	51 (50)	0.957 ± 0.075	0.671	78.41 ± 18.18	0.791
**6–10**	35 (34.3)	0.960 ± 0.039		79 ± 17.4	
**11–15**	16 (15.6)	0.963 ± 0.031		78.87 ± 12.97	
**Sport discipline**					
**CrossFit**	32 (31.3)	0.961 ± 0.087	**0.0016**	80.15 ± 14.17	**0.0013**
**CrossFit + cycling**	14 (13.7)	0.958 ± 0.036		84.92 ± 8.78	
**CrossFit + the Gym**	19 (18.6)	0.986 ± 0.024		86.52 ± 17.44	
**CrossFit + Nordic Walking**	11 (10.7)	0.932 ± 0.047		68.18 ± 17.26	
**CrossFit + running**	26 (25.4)	0.950 ± 0.042		72.23 ± 19.59	

Bold text denotes statistical significance.

**Table 2:** CCQ Total score and three domains analysis results: CCQ Symptoms, CCQ Functional state and CCQ Mental state outcomes with statistical evaluation.

	General Characteristics	CCQ Total Score		CCQ Symptoms (Items 1, 2, 5, 6)		CCQ Functional State (Items 7, 8, 9, 10)		CCQ Mental State (Items 3, 4)	
	n (%)	Mean ± SD	*p*	Mean ± SD	*p*	Mean ± SD	*p*	Mean ± SD	*p*
**Total**	102 (100)	0.94 ± 0.75		1.15 ± 0.93		0.75 ± 0.8		0.89 ± 1.06	
**Gender**									
**Female**	54 (52.9)	1.14 ± 0.84	**0.0092**	1.32 ± 1.04	0.0855	1.03 ± 0.90	**0.0002**	0.98 ± 1.05	0.186293
**Male**	48 (47)	0.71 ± 0.57		0.96 ± 0.77		0.42 ± 0.50		0.79 ± 1.08	
**Age (years)**									
**20–29**	39 (38.2)	0.94 ± 0.86	0.6963	1.24 ± 1.08	0.6432	0.71 ± 0.84	0.6535	0.79 ± 1.04	0.2322
**30–39**	28 (27.4)	1.02 ± 0.77		1.25 ± 0.91		0.82 ± 0.81		0.94 ± 1.05	
**40–49**	25 (24.5)	0.78 ± 0.52		1.01 ± 0.74		0.58 ± 0.52		0.76 ± 1.04	
**50–60**	10 (9.7)	1.1 ± 0.80		0.90 ± 0.80		1.12 ± 1.11		1.45 ± 1.21	
**BMI (kg/m^2^)**									
**18.5–24.9**	57 (55.8)	0.98 ± 0.73	0.3937	1.15 ± 0.95	0.9786	0.82 ± 0.79	0.1763	0.94 ± 1.06	0.3539
**25.0–29.9**	45 (44.1)	0.89 ± 0.78		1.15 ± 0.92		0.65 ± 0.81		0.82 ± 1.07	
**Training history (years)**									
**1–5**	51 (50)	0.83 ± 0.63	0.5498	1.06 ± 0.79	0.8192	0.64 ± 0.63	0.7786	0.71 ± 1.02	**0.0381**
**6–10**	35 (34.3)	1.10 ± 0.92		1.32 ± 1.18		0.85 ± 0.92		1.18 ± 1.09	
**11–15**	16 (15.6)	0.93 ± 0.68		1.06 ± 0.73		0.85 ± 0.98		0.81 ± 1.06	
**Sport disciplines**									
**CrossFit**	32 (31.3)	0.82 ± 0.64	**0.0002**	1.02 ± 0.74	**0.0071**	0.56 ± 0.65	**0.0001**	0.93 ± 1.18	0.2066
**CrossFit + cycling**	14 (13.7)	0.74 ± 0.72		0.92 ± 0.97		0.53 ± 0.63		0.78 ± 0.93	
**CrossFit + the Gym**	19 (18.6)	0.48 ± 0.43		0.72 ± 0.69		0.28 ± 0.33		0.39 ± 0.48	
**CrossFit + Nordic Walking**	11 (10.7)	1.49 ± 0.57		1.45 ± 0.71		1.77 ± 0.92		1.00 ± 1.14	
**CrossFit + running**	26 (25.4)	1.29 ± 0.89		1.63 ± 1.15		1.00 ± 0.82		1.21 ± 1.17	

Bold text denotes statistical significance.

**Table 3:** Results of the assessment of the severity of dyspnea according to the mMRC questionnaire with a statistical evaluation.

	mMRC (Grade 1)	mMRC (Grade 2)	mMRC (Grade 3)	*p*-Value
	n (%)	n (%)	n (%)	
Total	65 (63.72)	31 (30.39)	6 (5.88)	
Gender				
Female	31 (57.41)	18 (33.33)	5 (9.26)	0.195
Male	34 (70.83)	13 (27.08)	1 (2.08)	
Age (years)				
20–29	25 (64.10)	12 (30.77)	2 (5.13)	0.307
30–39	17 (60.71)	9 (32.14)	2 (7.14)	
40–49	19 (76.00)	6 (24.00)	0 (0)	
50–60	4 (40.00)	4 (40.00)	2 (20.00)	
BMI (kg/m^2^)				
18.5–24.9	37 (64.91)	16 (28.07)	4 (7.02)	0.763
25.0–29.9	28 (62.22)	15 (33.33)	2 (4.44)	
Training history (years)				
1–5	35 (68.63)	14 (27.45)	2 (3.92)	0.595
6–10	20 (57.14)	13 (37.14)	2 (5.71)	
11–15	10 (62.50)	4 (25.00)	2 (12.50)	
Sport disciplines				
CrossFit	22 (68.75)	9 (28.13)	1 (3.13)	0.198
CrossFit + cycling	7 (50.00)	5 (35.71)	2 (14.29)	
CrossFit + the Gym	17 (89.47)	2 (10.53)	0 (0)	
CrossFit + Nordic Walking	5 (45.45)	5 (45.45)	1 (9.09)	
CrossFit + running	14 (53.85)	10 (38.46)	2 (7.69)	

## Data Availability

The datasets used and/or analyzed during the current study are available from the corresponding author on reasonable request.
